# Deep learning body-composition analysis of clinically acquired CT-scans estimates creatinine excretion with high accuracy in patients and healthy individuals

**DOI:** 10.1038/s41598-022-13145-w

**Published:** 2022-05-30

**Authors:** Tobias T. Pieters, W. B. Veldhuis, P. Moeskops, B. D. de Vos, M. C. Verhaar, S. Haitjema, A. D. R. Huitema, M. B. Rookmaaker

**Affiliations:** 1 Department of Nephrology and Hypertension, University Medical Center Utrecht, Utrecht University, Room F03.225, Heidelberglaan 100, 3584 CX Utrecht, The Netherlands; 2 Department of Radiology, University Medical Center Utrecht, Utrecht University, Heidelberglaan 100, 3584 CX Utrecht, The Netherlands; 3 Quantib-U, Padualaan 8, 3584 CH Utrecht, The Netherlands; 4 Department of Biomedical Engineering and Physics, University Medical Center Amsterdam, Meibergdreef 9, 1105 AZ Amsterdam, The Netherlands; 5 Central Diagnostic Laboratory, University Medical Center Utrecht, Utrecht University, Heidelberglaan 100, 3584 CX Utrecht, The Netherlands; 6 Department of Pharmacy and Pharmacology, Netherlands Cancer Institute, Plesmanlaan 121, 1066 CX Amsterdam, The Netherlands; 7 Department of Pharmacology, Princes Maxima Centrum Utrecht, Heidelberglaan 25, 3584 CS Utrecht, The Netherlands; 8 Department of Clinical Pharmacy, University Medical Center Utrecht, Utrecht University, Heidelberglaan 100, 3584 CX Utrecht, The Netherlands

**Keywords:** Medical research, Nephrology, Mathematics and computing

## Abstract

Assessment of daily creatinine production and excretion plays a crucial role in the estimation of renal function. Creatinine excretion is estimated by creatinine excretion equations and implicitly in eGFR equations like MDRD and CKD-EPI. These equations are however unreliable in patients with aberrant body composition. In this study we developed and validated equations estimating creatinine production using deep learning body-composition analysis of clinically acquired CT-scans. We retrospectively included patients in our center that received any CT-scan including the abdomen and had a 24-h urine collection within 2 weeks of the scan (n = 636). To validate the equations in healthy individuals, we included a kidney donor dataset (n = 287). We used a deep learning algorithm to segment muscle and fat at the 3rd lumbar vertebra, calculate surface areas and extract radiomics parameters. Two equations for CT-based estimate of RenAl FuncTion (CRAFT 1 including CT parameters, age, weight, and stature and CRAFT 2 excluding weight and stature) were developed and compared to the Cockcroft-Gault and the Ix equations. CRAFT1 and CRAFT 2 were both unbiased (MPE = 0.18 and 0.16 mmol/day, respectively) and accurate (RMSE = 2.68 and 2.78 mmol/day, respectively) in the patient dataset and were more accurate than the Ix (RMSE = 3.46 mmol/day) and Cockcroft-Gault equation (RMSE = 3.52 mmol/day). In healthy kidney donors, CRAFT 1 and CRAFT 2 remained unbiased (MPE = − 0.71 and − 0.73 mmol/day respectively) and accurate (RMSE = 1.86 and 1.97 mmol/day, respectively). Deep learning-based extraction of body-composition parameters from abdominal CT-scans can be used to reliably estimate creatinine production in both patients as well as healthy individuals. The presented algorithm can improve the estimation of renal function in patients who have recently had a CT scan. The proposed methods provide an improved estimation of renal function that is fully automatic and can be readily implemented in routine clinical practice.

## Introduction

Assessment of daily creatinine excretion plays a crucial role in the evaluation of renal function and is used to extrapolate findings in urinary portions to daily excretions^1–4^. Although 24-h urine collection is considered the gold standard to assess daily creatinine excretion, this method is burdensome, not always possible and prone to collection errors^5^. Therefore, it is often estimated in renal function formulas such as the Cockcroft-Gault, CKD-EPI, and Ix equation, the latter developed as a creatinine clearance equation based on the original CKD-EPI cohorts^6–8^. These equations were derived in datasets where surrogates of body composition (i.e. age, sex, weight, and race) were used to estimate either the measured glomerular filtration rate, or the measured creatinine clearance. However, these equations may be unreliable in patients with aberrant body composition^9^.

Creatinine is a breakdown product predominantly formed in the muscles, which distributes in total body water and is excreted by the kidneys^10–12^. Conventional equations that estimate creatinine excretion are constructed using surrogate markers of muscle mass (e.g. age, gender, weight, and race). However, these equations have mostly been developed and validated in patients visiting the outpatient clinic and outliers such as patients with aberrant muscle mass were excluded^6,8^. In patients with aberrant muscle mass, the ability of surrogate markers to correctly estimate creatinine excretion is severely hampered and a direct measurement of body composition may be more accurate.

Clinically acquired CT-scans can be used to acquire body composition estimates such as muscle and fat volume^13^. The cross-sectional muscle area at the L3 level has a strong association with total body muscle volume^14^. Deep learning algorithms have been developed that automatically select L3 and segment the muscles, which provides the opportunity to incorporate these measurements in routine clinical care and large scientific studies^13^. These algorithms have shown to provide reliable automated segmentations of body composition at the L3 level compared to manual segmentation by experts^15,16^. Furthermore, these algorithms can extract radiomics parameters which provide additional parameters of body composition based on the radiation attenuation (i.e. distribution of Hounsfield units) that cannot easily be quantified in a reproducible way by the human eye^17^. Since abdominal CT-scans are increasingly performed in routine care, this provides the opportunity to automatically quantify body composition in large groups of patients without extra costs or burden to the patient.

The goal of this study was to create an equation that estimates creatinine production from automated image analysis of CT-scans that were made for other purposes, combined with traditional surrogate markers of body composition that are used in existing equations that estimate creatinine production such as weight and stature..

## Methods

### Patient population and data extraction

For the patient dataset, we included all patients that received a CT-scan of the abdomen and had a 24-h urine collection 2 weeks prior or after the CT-scan in the University Medical Center Utrecht (UMC Utrecht) between 2011 and 2019. Patients that were scanned more than once and had separate 24-h collections were considered as separate data entries if the scans were more than one week apart. In addition, we included a second dataset of kidney donors who received an abdominal CT-scan and multiple subsequent collections of 24-h urine as part of their routine screening between 2008 and 2020 in the UMC Utrecht. Kidney donors were also included if time between CT and 24-h urine creatinine excretion was more than two weeks, since they are likely to have stable muscle mass.

Pseudonymized CT-scans were extracted from the Research Image Archive of the UMC Utrecht. Patient and laboratory data were extracted from the Utrecht Patient Oriented Database (UPOD), an infrastructure of relational databases at out center^18^. Plasma and urine creatinine were measured by enzymatic colorimetric assay (Beckman Coulter, Brea, CA, USA). Clinical variables that were extracted from UPOD were age (years), weight (kg), stature (cm), and sex (male/female). Due to the retrospective nature of the study with collection of anonymized data, it was not possible to further specify reason for admittance.

### Selection of 24-h urine creatinine measurements

When plasma creatinine is not in steady-state, excretion of creatinine does not equal creatinine production due to build-up in the volume of distribution^19,20^. To correct for this, we calculated approximate increase or decrease of creatinine in the volume of distribution and excluded measurements exceeding 1.5 mmol/day, calculated by:

1 $$\Delta TBCr\left(\mathrm{mmol}/\mathrm{day}\right)=\Delta PCr\left(\frac{\mathrm{mmol}}{\mathrm{L}}/\mathrm{day}\right)*VD(\mathrm{L})$$

where ΔTBCr is the daily change in total body creatinine, ΔPCr is the daily change in plasma creatinine, and VD is the volume of distribution.

As a measure of volume of distribution we used 60% of bodyweight, an estimate of total body water^10–12^. In addition, we excluded measurements with biologically implausible values (< 3 mmol and > 30 mmol/day). If a patient had multiple consecutive urine measurements, the mean of these values was used.

### Feature extraction from CT-scans and calculation of radiomics parameters

We included any CT-scan covering the L3 segment, including any variation of intravenous contrast. CT examinations were obtained with multidetector scanners: Mx8000 IDT 16, Brilliance 64, iQon Spectral or Brilliance iCT; all from Philips Medical Systems, Cleveland, OH, USA. Exposure settings (range: 35–190 mAs and 80–120 kVp) were adjusted to patient size.

The deep learning algorithm (Quantib Body Composition version 0.2.1.) has been developed previously in separate datasets and is described previously and is available^13,21^. Briefly, the L3 slices were automatically identified using a convolutional neural network with a regression output. The network was trained to estimate, for every slice in the image, its proximity to the L3 vertebra. During automated processing, the slice estimated by the network as closest to the craniocaudal middle of L3 was labelled as the L3 slice. Second, at the estimated L3 level, automated segmentation into areas (visceral/subcutaneous fat, psoas/long spine/abdominal wall muscles) was performed using a dilated residual network trained using a cross-entropy loss function. Both networks were implemented using PyTorch1.6. To increase robustness of body composition measurements, image segmentation was performed for five slices of 5 mm around the L3 slice, i.e., two slices below and two slices above. Therefore, the resulting segmentation areas were averaged over a range 2.5 cm. The algorithm segmented muscle (psoas, long spine, and abdominal wall muscles) and fat compartments (subcutaneous and visceral), and further segmentation was performed using threshold values for Hounsfield Units (HU) for muscle (> − 15 HU)^22^. Furthermore, radiomics parameters were calculated from the HU distribution of all muscle and fat compartments. Radiomics parameters that were calculated and used for variable selection were: percentiles (10th, 25th, 75th, 90th), kurtosis, skewness, mean, median, standard deviation, minimum, and maximum. After segmentation by the algorithm, slices were manually checked for sufficient quality of the scan (e.g. no artefacts and/or cut-off muscle).

### Handling of missing data, construction and validation of the equations

The patient dataset was split in a development and validation set (random split 60%/40%). Missing data was imputed separately in the development (weight 22%, stature 9%), validation (weight 19%, stature 7%), and kidney donor (weight < 1%, stature < 1%) set (m = 10)^23^. All clinical and radiomics variables were used as predictors and both weight and stature were imputed using predictive mean matching.

We used the LASSO algorithm to perform the first variable selection in the development dataset^24^. The LASSO algorithm is specifically designed to handle datasets with large amount of predictors and has previously been used for variable selection in datasets containing radiomics variables^25,26^. Variables that were selected in more than half of the imputed sets were further selected using least squares regression with backwards selection and tenfold cross-validation^27^. The equation with the lowest cross-validated mean RMSE was selected. Model coefficients were calculated using least squares regression and averaged over the 10 imputed datasets. We selected two equations, one where all parameters were considered (CRAFT 1) and one where weight and stature were left out (CRAFT 2).

Performance was compared to two previously published formulas, the Cockcroft-Gault formula and the Ix^7,8^. The Ix formula was calculated without the variable race, since this is not routinely recorded for patients in the Netherlands. We did not perform sample size calculation prior to inclusion, since there is no validated method to perform this when using LASSO for feature selection. We evaluated the risk of introducing bias by overfitting by comparing the results of our equations in the development dataset versus the validation dataset, which were similar.

### Statistics

We assessed bias with the Mean Prediction Error (MPE), precision with the R^2^-value of least squares regression, and accuracy with the RMSE and the number of predictions that fell within 15% (p15) or 30% (p30) of the outcome. We computed confidence intervals using the combined variance of bootstrap (1000x) and multiple imputation. For p15 and p30, we used the normal approximation. Relative accuracy of equations was evaluated based on the 95% CI of the ΔRMSE with CRAFT 1 as reference^28^.

All calculations and statistical analyses were executed using R statistics 3.5.1.

### Ethics

This study was performed according to the declaration of Helsinki and the ethical guidelines of our institution. The research protocol was approved by the institutional review board of the UMC Utrecht and they waived the need for informed consent (protocol number 20-247/C). The clinical and research activities being reported are consistent with the Principles of the Declaration of Istanbul as outlined in the 'Declaration of Istanbul on Organ Trafficking and Transplant Tourism'. Pseudonymized data were used for this study. Data collection and handling was conducted in accordance with European privacy legislation (GDPR).

## Results

### Characteristics of patients and healthy kidney donors

For the patient dataset, we included 702 abdominal CT-scans. We excluded 19 scans because of insufficient scan quality and 16 scans with segmentation errors (Supplemental Table 1, Supplemental Fig. 1). Finally, we excluded 31 scans that were performed within a week of each other in the same patient, therefore including 636 scans for development (n = 370) and validation (n = 266). Furthermore, we included 287 kidney donors who received a CT-scan and 2–3 consecutive 24-h urine collections in our center. In the kidney donor dataset, all scans were of sufficient quality, thus none were excluded (Table 1).

**Table 1:** Patient characteristics in the development, validation, and kidney donor data sets.

Characteristic	Development	Validation	Kidney donors
Number of patients	370	266	287
Age (years)	55 ± 15	55 ± 15	54 ± 12
Sex (male/female)	208/162	149/117	112/175
Weight (kg)	78 ± 18	79 ± 18	76 ± 12
Stature (cm)	173 ± 10	173 ± 10	172 ± 13
BMI (kg/m^2^)	26 ± 6	26 ± 5	25 ± 3
BSA (m^2^)	1.91 ± 0.23	1.91 ± 0.25	1.89 ± 0.19
In-patient/out-patient	120/250	96/170	0/287
Urine creatinine (mmol/day)	11 ± 4	11 ± 4	12 ± 5
Psoas volume > − 15HU (cm^2^)	19 ± 7	18 ± 7	18 ± 6
Psoas mean RA > − 15HU (HU)	44 ± 9	44 ± 9	48 ± 6
Long spine volume > − 15HU (cm^2^)	44 ± 10	43 ± 12	46 ± 10
Long spine mean RA > − 15HU (HU)	38 ± 11	37 ± 12	44 ± 9
Abdominal wall volume > − 15HU (cm^2^)	68 ± 20	67 ± 20	66 ± 18
Abdominal wall mean RA > − 15HU (HU)	34 ± 11	33 ± 11	36 ± 8

Values are expressed as mean ± SD.

*BMI* body mass index, *BSA* body surface area, *RA* radiation attenuation, *HU* hounsfield units.

Among the combined datasets (n = 923), 49% was female, mean age was 55 (SD ± 14) years, mean BMI was 26 (SD ± 4.5) and mean creatinine excretion was 12 (SD ± 4.1) mmol/day. Apart from sex, clinical characteristics did not differ significantly between the datasets. The intraindividual variability of urine collections in kidney donors, defined as the mean standard deviation of 2–3 consecutive collections calculated for individual donors, was 1.47 mmol/day.

### Association of muscle volume and clinical variables with creatinine excretion

**Figure 1:**
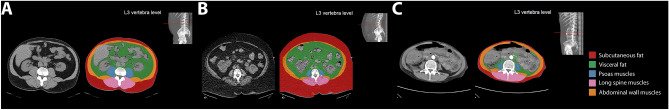
Examples of segmentation by the algorithm in a patient with average (**A**), obese (**B**), and cachectic (**C**) body composition. Red is subcutaneous fat, green is visceral fat, blue are the psoas muscles, purple are the long spine muscles and orange are the abdominal wall muscles.

**Figure 2:**
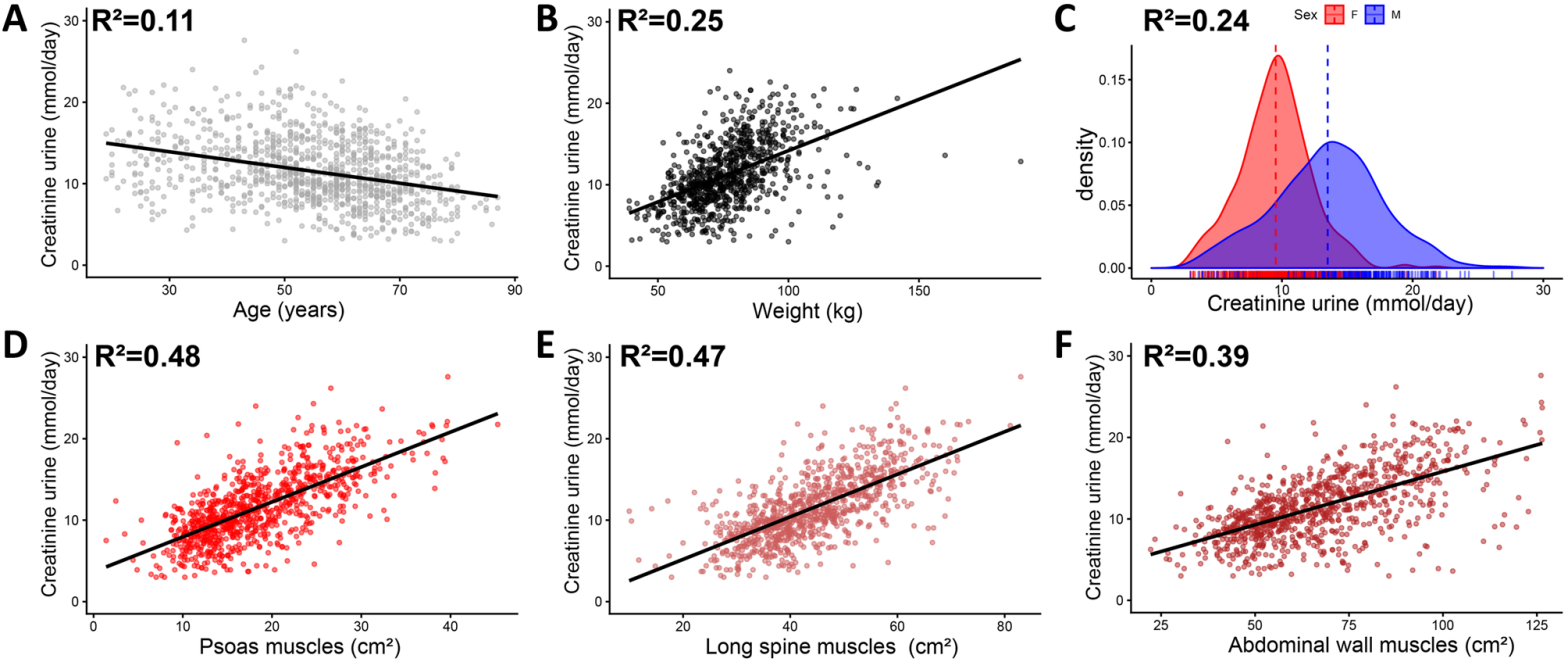
Plots comparing muscle surface areas and clinical parameters with urinary creatinine excretion. (**A**–**C**) Association with clinical characteristics age (**A**), weight (**B**), and Sex (**C**). (**D**–**F**) Association between muscle surface areas (segmented > − 15HU) from the psoas muscles (**D**), long spine muscles (**E**), and abdominal wall muscles (**F**). Solid regression lines in dot plots are calculated with ordinary least squares regression. The probability density was calculated with kernel density estimation and shows excretion in the female (F, red colour) and male sex (M, blue colour). The dashed line represents the mean of the different groups. The R^2^-values were calculated with ordinary least squares regression.

Figure 1 shows examples of the result of the segmentation in varying body compositions. Surface areas of the muscles had a strong association with creatinine excretion in the combined cohorts (Fig. 2) (R^2^ = 0.48, 0.47 and 0.39 respectively). Traditional clinical variables (e.g. weight, age, sex) also associated with creatinine excretion, although the correlation was less strong (Fig. 2) (R^2^ = 0.11, 0.25 and 0.24 respectively).

### Development of equations using clinical and radiomics parameters

We developed two equations, one where both clinical and radiomics variables were considered (CRAFT 1) and one where weight and stature were not used (CRAFT 2). After multiple imputation (Supplemental Fig. 2), we performed initial variable selection with LASSO for CRAFT 1 (214 variables considered) and CRAFT 2 (212 variables considered). We selected all variables that were picked in more than half of the imputed datasets (11 variables for both equations). Further selection with tenfold cross-validation and backwards selection on these variables resulted in 10 variables for CRAFT 1 and 11 variables for CRAFT 2 (Supplemental Fig. 3, Supplemental Table 2).

### High accuracy of CT-based equations in patient validation dataset

**Figure 3:**
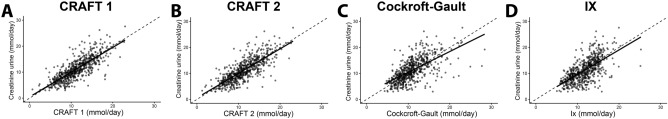
Plots comparing CRAFT 1 (**A**), CRAFT 2 (**B**), Cockcroft-Gault (**C**), and Ix equation (**D**) to creatinine excretion in the validation dataset. Solid regression lines in dot plots are calculated with ordinary least squares regression. The dashed line is the line of identity.

Both equations showed excellent performance in the patient validation dataset (Table 2, Fig. 3). CRAFT 1 and CRAFT 2 showed little bias (MPE = 0.18 mmol/day, and MPE = 0.16 mmol/day, respectively), whilst precision was moderate (R^2^ = 0.63 and R^2^ = 0.61, respectively). Both CRAFT 1 (RMSE = 2.68 mmol/day, p15 = 56%, p30 = 81%) and CRAFT 2 (RMSE = 2.78 mmol/day, p15 = 49%, p30 = 80%) displayed excellent accuracy. The new equations outperformed the Cockcroft-Gault (MPE = -0.16 mmol/day, R^2^ = 0.40, RMSE = 3.52 mmol/day, p15 = 40%, p30 = 77%) and Ix equation (MPE = 0.84 mmol/day, R^2^ = 0.42, RMSE = 3.46, p15 = 46%, p30 = 71%). Both the Cockcroft-Gault equation (compared to CRAFT 1 ΔRMSE [95% CI] 0.84 [0.47; 1.21]) and Ix equation (compared to CRAFT 1 ΔRMSE [95% CI] 0.79 [0.41; 1.16]) where less accurate compared to CRAFT 1, whilst CRAFT 1 and CRAFT 2 had more or less similar accuracy (ΔRMSE [95% CI] 0.10 [− 0.01; 0.20]. The difference between CRAFT 1 and CRAFT 2 and the Ix and Cockcroft–Gault equations was more pronounced in in-patients than in out-patients (Supplemental Table 3).

**Table 2:** Equation performance in validation dataset (n = 266).

Formula	Bias	Precision	Accuracy			
	MPE [95% CI]	R^2^ [95% CI]	RMSE [95% CI]	MAPE [95% CI]	P15 [95% CI]	P30 [95% CI]
Cockcroft–Gault	− 0.16 [− 0.60; 0.27]	0.40 [0.28; 0.52]	3.52 [3.07; 3.96]	27.2% [22.7–31.7%]	40% [34%; 46%]	77% [72%; 82%]
Ix	0.84 [0.44; 1.25]	0.42 [0.31; 0.52]	3.46 [3.07; 3.85]	30.3% [25.2–35.5%]	46% [40%; 52%]	71% [66%; 77%]
CRAFT 1	0.18 [− 0.13; 0.50]	0.63 [0.53; 0.72]	2.68 [2.34; 3.01]	21.0% [17.7–24.2%]	56% [50%; 62%]	81% [76%; 85%]
CRAFT 2	0.16 [− 0.17; 0.48]	0.61 [0.50; 0.70]	2.78 [2.43; 3.12]	22.3% [18.8–25.8]	49% [44%; 55%]	80% [76%; 85%]

Confidence intervals were calculated with the combined variance of multiple imputation (10×) and bootstrap (1000×).

*MPE* mean prediction error (mmol/day), *MAPE* mean absolute percentage error, *RMSE* root mean squared error (mmol/day), *R2* the R2-value calculated with linear regression, *p15/p30* the percentage of points that fall within 15%/30% of the outcome.

### Low level of bias of CT-based based equations in healthy kidney donors

**Figure 4:**
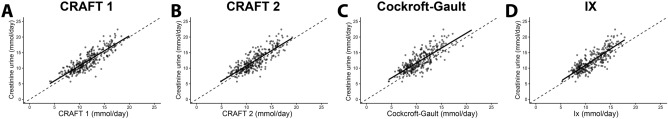
Plots comparing the CRAFT 1 (**A**), CRAFT 2 (**B**), Cockcroft-Gault (**C**), and Ix equation (**D**) to creatinine excretion in the kidney donor dataset. Solid regression lines in dot plots are calculated with ordinary least squares regression. The dashed line is the line of identity.

To ensure that the new equations were also unbiased in healthy individuals, we validated the equations in a group of kidney donors (Table 3, Fig. 4). Both CRAFT 1 and CRAFT 2 displayed slight negative bias compared to the patient validation cohort (− 0.71 mmol/day and − 0.73 mmol/day respectively), although precision was higher (R^2^ = 0.73 and R^2^ = 0.69, respectively). Furthermore, accuracy was better in both CRAFT 1 (RMSE = 1.86 mmol/day, p15 = 70%, p30 = 97%) and CRAFT 2 (RMSE = 1.97 mmol/day, p15 = 67%, p30 = 95%). CRAFT 1 and CRAFT 2 were more accurate than the Ix equation (MPE = − 1.08 mmol/day, R^2^ = 0.70, RMSE = 2.10, p15 = 63%, p30 = 97%) and the Cockcroft–Gault equation (MPE = − 1.66 mmol/day, R^2^ = 0.64, RMSE = 2.57, p15 = 47%, p30 = 90%). The Ix and Cockcroft-Gault equations were also less accurate than CRAFT 1, although the difference was less pronounced with the Ix equation (compared to CRAFT 1 ΔRMSE [95% CI] 0.24 [0.10; 0.38]) than with the Cockcroft-Gault equation (compared to CRAFT 1 ΔRMSE [95% CI] 0.71 [0.56; 0.87]). CRAFT 2 again had similar accuracy, although it was slightly less accurate (compared to CRAFT 1 ΔRMSE [95% CI] 0.11 [0.06; 0.17]).

**Table 3:** Equation performance in kidney donor dataset (n = 287).

Formula	Bias	Precision	Accuracy			
	MPE [95%CI]	R2 [95% CI]	RMSE [95% CI]	MAPE [95% CI]	P15 [95% CI]	P30 [95% CI]
Cockcroft–Gault	− 1.66 [− 1.88; − 1.43]	0.64 [0.57; 0.70]	2.57 [2.37; 2.76]	16.5% [15.3–17.7%]	47% [41%; 53%]	90% [86%; 93%]
Ix	− 1.08 [− 1.29; − 0.87]	0.70 [0.64; 0.75]	2.10 [1.92; 2.27]	13.1% [12.1–14.2%]	63% [57%; 68%]	97% [95%; 99%]
CRAFT 1	− 0.71 [− 0.91; − 0.51]	0.73 [0.67; 0.78]	1.86 [1.70; 2.01]	12.2%[11.1–13.3%]	70% [65%; 75%]	97% [94%; 99%]
CRAFT 2	− 0.73 [− 0.94; 0.52]	0.69 [0.63; 0.74]	1.97 [1.80; 2.14]	12.7% [11.6–13.8%]	67% [62%; 72%]	95% [93%; 98%]

Confidence intervals were calculated with the combined variance of multiple imputation (10×) and bootstrap (1000×).

*MPE* mean prediction error (mmol/day), *MAPE* mean absolute percentage error, *RMSE* root mean squared error (mmol/day), *R2* the R2-value calculated with linear regression, *p15/p30* the percentage of points that fall within 15%/30% of the outcome.

### Accuracy of equations at different levels of creatinine excretion

Next, we compared the accuracy at different tertiles of creatinine excretion (Supplemental Tables 4, 5). CRAFT 1 was the most accurate across all tertiles in the patient dataset. The difference in accuracy between CRAFT 1 and the Ix equation was the most pronounced in the lowest tertile, whilst the difference in accuracy between CRAFT 1 and the Cockcroft-Gault equation was highest in the highest tertile. In the kidney donor dataset, the difference in accuracy was more or less comparable across tertiles.

## Discussion

We demonstrated that deep learning-based analysis of abdominal CT scans at L3 can improve the estimation of creatinine excretion. We developed and validated two equations to estimate creatinine excretion based on clinical characteristics and measures of body composition. The equations showed excellent accuracy in an unselected group of patients with varying body compositions and in an additional group of healthy kidney donors. When compared with previously published equations, our equations improved the accuracy of the estimation of creatinine excretion especially in patients with relatively low or high creatinine excretion.

The strong correlation between body composition at L3 and creatinine excretion is in line with previous studies that examined body compartments at L3. Radiologic assessment of body composition at L3 has been shown to be an objective measure of obesity and muscle wasting in patients with end-stage liver disease awaiting transplantation^29^. The L3 skeletal muscle index has been shown to be a marker for sarcopenia and frailty in lung cancer patients^30^. Assessment of sarcopenia and changes in body composition have been shown to associate with outcomes after neoadjuvant chemotherapy in oesophageal cancer^31^. Furthermore, analysis of body composition may help in dosing chemotherapy and dosing contrast injection in contrast-enhanced CT^32,33^. We add to these observations that body composition analysis at L3 can be used to accurately estimate creatinine excretion.

The finding that our CT-based equations had the most benefit over clinical equations in the patient cohort is most likely explained by the fact that this cohort included a wide range of sick and/or hospitalized patients with altered body composition. This is further emphasized by the fact that our equations had the most benefit over the clinical equations in the lowest tertile of creatinine excretion in patients, where there are likely patients included with muscle wasting. Indeed, it has previously been shown that eGFR equations perform worse in hospitalized patients^9,34^. In addition, the Ix formula was developed and validated in cohorts were patients with liver disease, cancer, and muscle mass far from population norms were excluded, whilst we did not exclude patients based on these characteristics^8^. Our results indicate that in these patients where creatinine excretion is hard to estimate based on clinical variables, our equations may provide the most benefit.

Although the new equations use direct measures of muscle mass to predict creatinine excretion, there was still residual inaccuracy. This may be explained by several factors. Although we excluded patients with fluctuating serum creatinine, build-up of serum creatinine in the volume of distribution may still have affected creatinine excretion^19^. Furthermore, muscle volume and radiation attenuation at L3 may not be fully representative of total body muscle volume, especially in areas not represented such as the extremities. Finally, the residual inaccuracy may be the result of errors in the timed urine collections. This is illustrated by the mean standard deviation of creatinine excretion in kidney donors (1.47 mmol/day), which was close to the standard deviation of the prediction error of CRAFT 1 in the kidney donor set (RMSE = 1.86 mmol/day). This suggests that a large part of the residual inaccuracy may have been caused by collection errors.

Strengths of this study includes it large sample size and the use of direct measures of muscle volume and body composition to estimate creatinine excretion. Furthermore, this was a retrospective study in real-life clinical data where we did not exclude patients with aberrant muscle mass. We validated the formula in both patients and healthy individuals, further supporting its validity in diseased and healthy individuals. Since the analysis of the CT-scan is fully automated, it may be easily implemented in routine clinical care. Finally, with the use of direct measures of body composition variables that are not automatically available or less discrete such as race may no longer be needed in renal function formulas^35,36^.

The retrospective nature of this study may have led to selection bias, since we could only evaluate the in patients that received a CT-scan and a 24-h urine collection. However, the equations also performed well in kidney donors, indicating that the equations may be accurate in a broader group of patients. Since this is a single-center study, the equations should still be validated in other patient groups and other centers. We could not calculate the Ix equation with the race variable and used the Caucasian race as default, which may have decreased the accuracy of this equation in patients of other ethnic backgrounds. The lack of data on race also prohibited us from analysing whether a difference in muscle volume/density explains the difference in creatinine excretion between people of different ethnical backgrounds that has previously been observed. The use of a race variable in renal function formulas is however currently under debate, not only for ethical reasons but also because an increasing amount of people identify as being of mixed racial background^36^.

In the future, automated body composition analysis of abdominal CT-scans could be fully integrated in electronic health records. Automated reporting of our equations may be used to validate the result of the CKD-EPI and replace the laborious collection of 24-h urine with spot urine collections. This combination of automatically generated data from the radiology and laboratory department represents a trend in diagnostic medicine called integrated diagnostics, where multidisciplinary data is combined to provide the treating physician with lean and accurate information^37,38^.

In conclusion, we developed and validated equations that estimate creatinine production by adding automated deep learning body-composition analysis of clinically acquired CT-scans to traditional clinical characteristics. These equations were superior to previously published formulas, especially in patients with aberrant body composition. Addition of creatinine production to the results of CT scans may improve and individualize the estimation of renal function derived from plasma creatinine concentrations as compared to traditional renal function formulas.

## Supplementary Information

Supplementary Figures.

Supplementary Tables.
