# Supplementary material for: Deep learning body-composition analysis of clinically acquired CT-scans estimates creatinine excretion with high accuracy in patients and healthy individuals
Source: Sci Rep. 2022 May 30;12:9013. doi: 10.1038/s41598-022-13145-w (PMC9151677; doi:10.1038/s41598-022-13145-w)
Supplement: Supplementary file 1 — Supplementary Figures. [file 41598_2022_13145_MOESM1_ESM.docx]

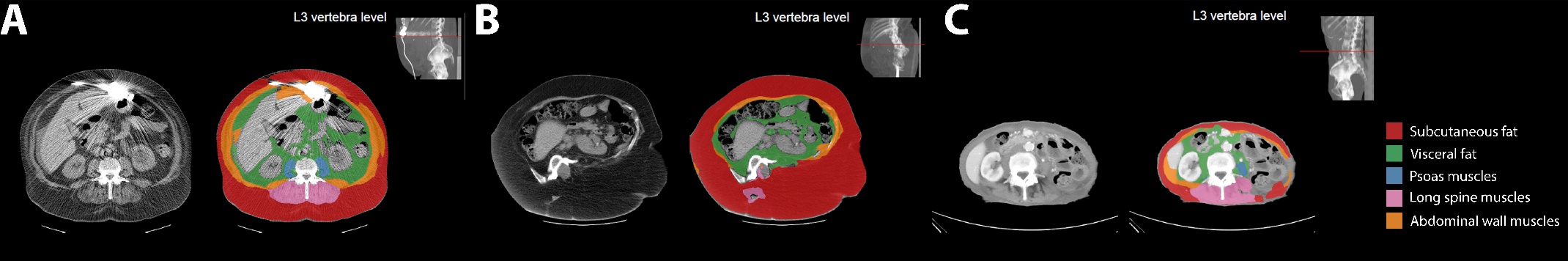


**Supplemental Figure 1.** Examples of scans that were excluded for final analyses. **(A)** Beam hardening artefact prevents accurate segmentation of abdominal muscles. **(B)** Severe scoliosis prevents accurate segmentation of muscles. **(C)** Severe cachexia prevents accurate segmentation of muscles.


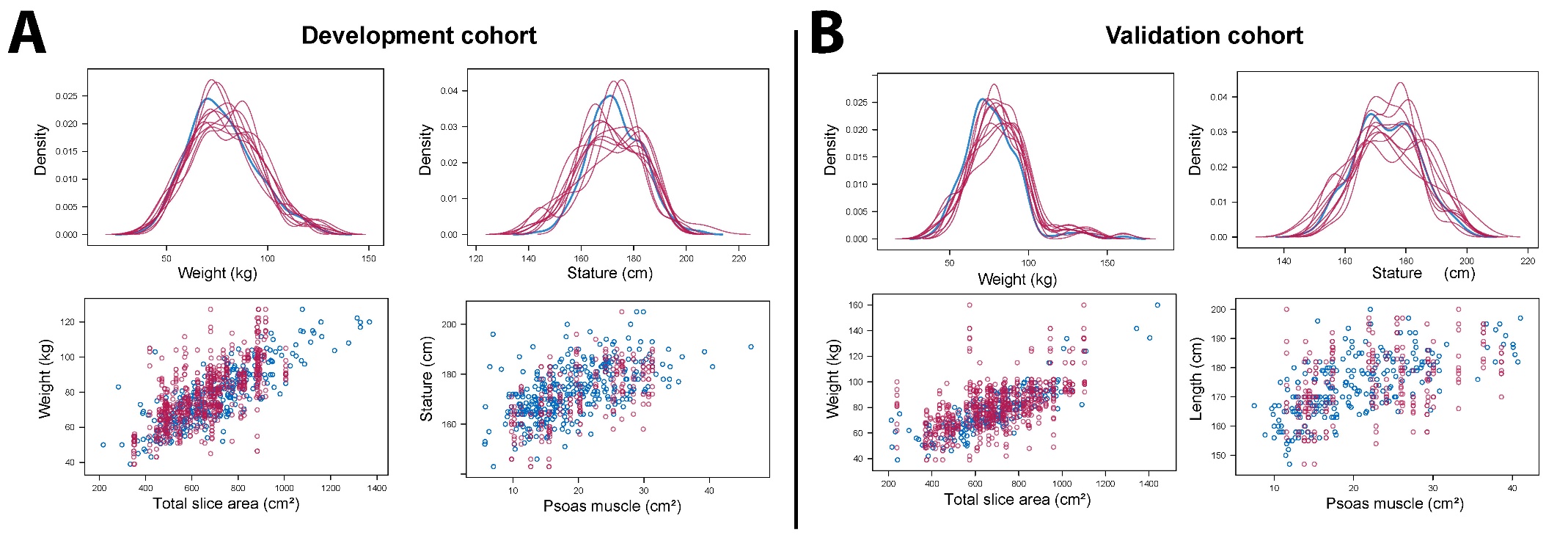


**Supplemental Figure 2.** Multiple imputation of weight and stature in the imputed datasets (m=10, pink) compared to the original dataset (blue) in the development (**A**) and validation (**B**) dataset. Multiple imputation in the kidney donor data set is not shown, since this encompasses 1-2 imputations. The upper plots display kernel density plots. The bottom plots display dot plots of weight and stature and the characteristic with the highest correlation with these variables (total slice area and surface area of the psoas muscle respectively).


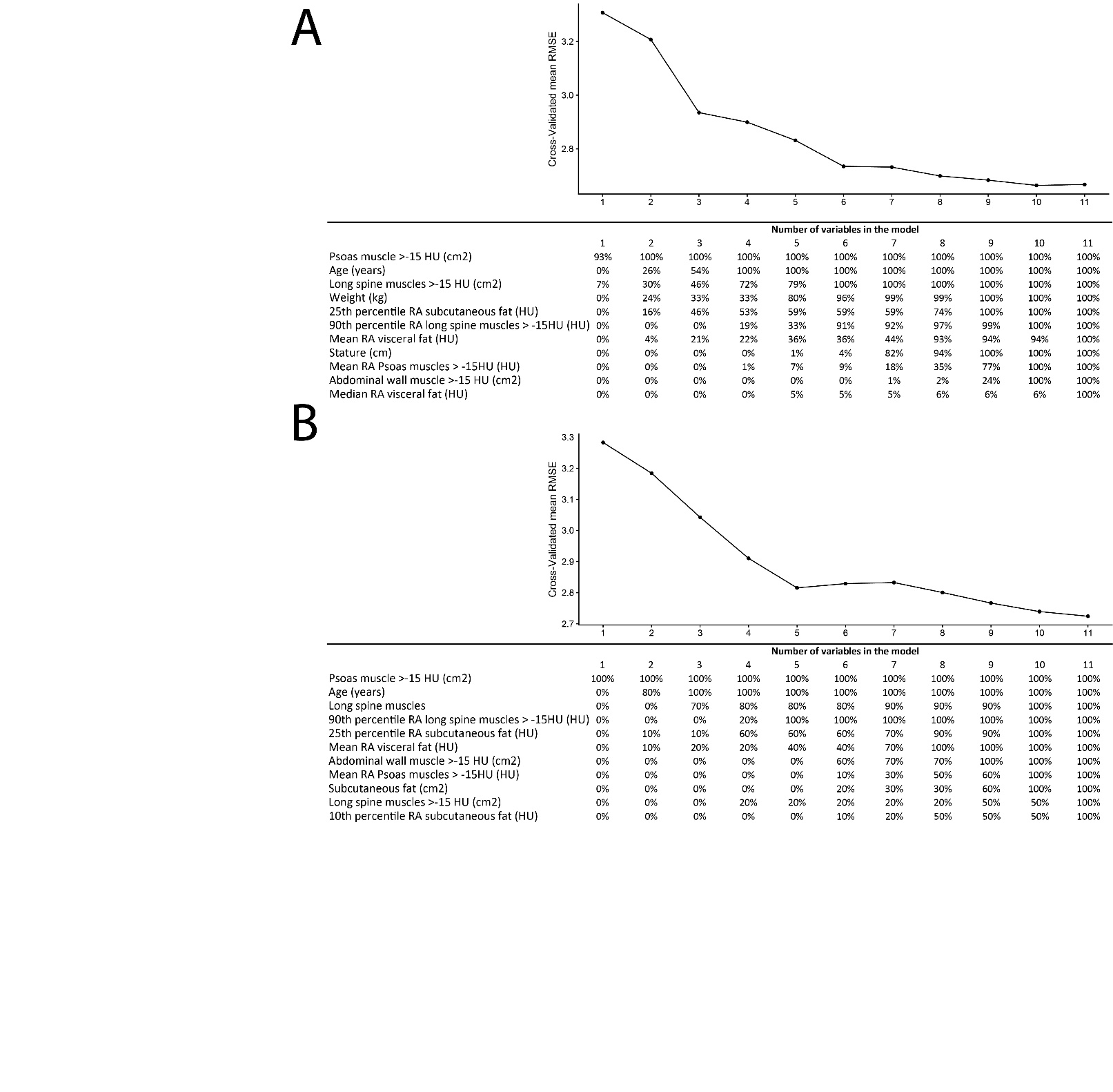


**Supplemental figure 3.** Cross-validation with backwards selection was used for variable selection for CRAFT 1 **(A)** and CRAFT 2 **(B)** in the development cohort. The top graphs displays the mean cross-validated RMSE on the y-axes and the number of variables included in the equation on the x-axes. The tables on the bottom displays the percentage of times variables were in the equation across the Cross-validated and imputed datasets when a certain amount of variables was selected for the equation.
