# Supplementary material for: Deep learning body-composition analysis of clinically acquired CT-scans estimates creatinine excretion with high accuracy in patients and healthy individuals
Source: Sci Rep. 2022 May 30;12:9013. doi: 10.1038/s41598-022-13145-w (PMC9151677; doi:10.1038/s41598-022-13145-w)
Supplement: Supplementary file 2 — Supplementary Tables. [file 41598_2022_13145_MOESM2_ESM.docx]

| **Supplemental table 1.** Reason for exclusion of scans | |  |
| --- | --- | --- |
| **Reason for exclusion** |  | |
| Severe scoliosis | 5 | |
| Artefact | 5 | |
| Partial FOV | 9 | |
| Segmentation error | 11 | |
| Level selected other than L3 | 5 | |

| **Supplemental Table 2.** Coefficients with 95% CI of models CRAFT 1 and CRAFT 2 | | |
| --- | --- | --- |
| **Coefficient** | **CRAFT 1** | **CRAFT 2** |
| Intercept | -12.6170 [-18.3728 ; -6.8613] | -5.8913 [-9.5162 ; -2.2663] |
| Psoas muscle >-15 HU (cm2) | 0.1490 [0.0760 ; 0.2220] | 0.1863 [0.1163 ; 0.2564] |
| Long spine muscles (cm2) |  | 0.0744 [0.0071 ; 0.1559] |
| Long spine muscles >-15 HU (cm2) | 0.0791 [0.0367 ; 0.1214] | 0.0355 [-0.0470 ; 0.1181] |
| 90^th^ percentile RA long spine muscles > -15HU (HU) | 0.0345 [0.0078 ; 0.0613] | 0.0561 [-0.00003 ; 0.1123] |
| Mean RA Psoas muscles > -15HU (HU) | 0.0485 [0.0023 ; 0.0946] | 0.0361 [-0.0156 ; 0.0879] |
| 25^th^ percentile RA subcutaneous fat (HU) | -0.0307 [-0.0573 ; -0.0040] | -0.0790 [-0.2020 ; 0.0439] |
| 10^th^ percentile RA subcutaneous fat (HU) |  | 0.0537 [-0.071 ; 0.1786] |
| Mean RA visceral fat (HU) | -0.0227 [-0.0416 ; -0.0038] | -0.0228 [-0.0423 ; -0.0034] |
| Abdominal wall muscle >-15 HU (cm2) | 0.0174 [-0.0109 ; 0.0457] | 0.0259 [-0.0020 ; 0.0537] |
| Subcutaneous fat (cm2) |  | 0.0029 [-0.0008 ; 0.0065] |
| Age (years) | -0.0570 [-0.0780 ; -0.0360] | -0.0578 [-0.0804 ; -0.0351] |
| Stature (cm) | 0.0442 [0.0103 ; 0.0782] |  |
| Weight (kg) | 0.0334 [0.0098 ; 0.0570] |  |
| Data are shown as unstandardized coefficients [95% confidence interval]. Estimates were averaged over imputed datasets, 95% confidence interval was calculated with the combined variance of bootstrap (1000x) and multiple imputation (m=10 datasets). Weight and stature were excluded from model building for CRAFT 2. | | |

| **Supplemental Table 3.** Relative accuracy in the validation cohort, difference between in-patients and out-patients | | | | |
| --- | --- | --- | --- | --- |
|  | **Clinical patients (n= 96)** | | **Outhouse patients (n= 170)** | |
| **Formula** | **RMSE** | **ΔRMSE** | **RMSE** | **ΔRMSE** |
| Cockcroft-Gault | 3.59 [2.75-4.42] | 0.95 [0.17 – 1.74] | 3.44 [2.94-3.94] | 0.73 [0.36-1.10] |
| Ix | 3.89 [3.24-4.54] | 1.26 [0.56 – 1.96] | 3.14 [2.67-3.62] | 0.43 [0.05-0.82] |
| CRAFT 1 | 2.63 [2.17-3.10] | ref | 2.71 [2.45-3.17] | ref |
| CRAFT 2 | 2.78 [2.32-3.25] | 0.15 [0.00-0.30] | 2.77 [2.30-3.25] | 0.07 [-0.08-0.21] |
| **RMSE**= Root Mean Squared Error, **ΔRMSE**= the difference in RMSE with CRAFT 1 (ref). Confidence intervals were calculated as the combined variance of multiple imputation (10x) and bootstrap (1000x). | | | | |

| **Supplemental Table 4.** Relative accuracy at different levels of creatinine in the patient validation cohort | | | | | | |
| --- | --- | --- | --- | --- | --- | --- |
|  | **Lowest tertile** | | **Middle tertile** | | **Highest tertile** | |
| **Formula** | **RMSE** | **ΔRMSE** | **RMSE** | **ΔRMSE** | **RMSE** | **ΔRMSE** |
| Cockcroft-Gault | 3.78 [2.98 ; 4.59] | 0.59 [-0.04 ; 1.22] | 2.81 [1.84 ; 3.78] | 0.98 [0.03 ; 1.93] | 3.88 [3.26 ; 4.50] | 1.11 [0.67 ; 1.55] |
| Ix | 4.56 [3.93 ; 5.19] | 1.37 [0.70 ; 2.04] | 2.33 [0.50 ; 1.39] | 0.50 [-0.39 ; 1.39] | 3.12 [2.59 ; 3.65] | 0.35 [-0.11 ; 0.81] |
| CRAFT 1 | 3.19 [2.53 ; 3.86] | ref | 1.83 [1.55 ; 2.11] | ref | 2.77 [2.22 ; 3.32] | ref |
| CRAFT 2 | 3.30 [2.59 ; 4.01] | 0.11 [-0.10 ; 0.32] | 1.98 [1.71 ; 2.25] | 0.15 [0.01 ; 0.29] | 2.89 [2.31 ; 3.39] | 0.08 [-0.06 ; 0.22] |
| **RMSE**= Root Mean Squared Error, **ΔRMSE**= the difference in RMSE with CRAFT 1 (ref). Confidence intervals were calculated as the combined variance of multiple imputation (10x) and bootstrap (1000x). Tertiles were stratified by sex: males (10.7, 14.5 mmol/day), females (7.6, 9.9 mmol/day) | | | | | | |

| **Supplemental Table 5.** Relative accuracy at different levels of creatinine in the kidney donor cohort | | | | | | |
| --- | --- | --- | --- | --- | --- | --- |
|  | **Lowest tertile** | | **Middle tertile** | | **Highest tertile** | |
| **Formula** | **RMSE** | **ΔRMSE** | **RMSE** | **ΔRMSE** | **RMSE** | **ΔRMSE** |
| Cockcroft-Gault | 1.76 [1.55 ; 1.98] | 0.16 [-0.12 ; 0.43] | 2.48 [2.18 ; 2.77] | 0.88 [0.61 ; 1.15] | 3.32 [2.87 ; 3.59] | 0.95 [0.71 ; 1.19] |
| Ix | 1.38 [1.18 ; 1.60] | -0.22 [-0.43 ; 0.01] | 1.48 [1.31 ; 1.65] | -0.11 [-0.33 ; 0.11] | 3.00 [2.71 ; 3.30] | 0.72 [0.52 ; 0.93] |
| CRAFT 1 | 1.61 [1.33 ; 1.89] | ref | 1.59 [1.36 ; 1.83] | ref | 2.28 [2.00 ; 2.56] | Ref |
| CRAFT 2 | 1.66 [1.41 ; 1.90] | 0.05 [-0.05 ; 0.15] | 1.67 [1.41 ; 1.92] | 0.07 [-0.03 ; 0.18] | 2.46 [2.15 ; 2.77] | 0.18 [0.09 ; 0.27] |
| **RMSE**= Root Mean Squared Error, **ΔRMSE**= the difference in RMSE with CRAFT 1 (ref). Confidence intervals were calculated as the combined variance of multiple imputation (10x) and bootstrap (1000x). Tertiles were stratified by sex: males (13.9, 16.5 mmol/day), females (9.4, 11.0 mmol/day) | | | | | | |
